# Mr. Wilkinson in Answer to Mr. Cuming

**Published:** 1805-07-01

**Authors:** G. Wilkinson

**Affiliations:** Sunderland


### *To the Editors of the Medical and Physical Journal.*

Gentlemen,

On the first perusal of Mr. Cuming's reply to my observations on his controversy with Dr. Scott,* I thought it most consistent, as the discussion, from the shape it had assumed, appeared susceptible of no other effort than that of recrimination and personal remark, to consign the matter of debate to oblivion, and to leave to the slow working hand of time the task of ameliorating the petty animosities to which it had given rise. I am conscious that in every instance where individuals are occupied in the pursuits of science, should they reciprocally, by too sanguine an enforcement of their peculiar opinions, give birth to irritability of feeling, a series of impassioned anathemas might occur, unconnected with sobriety of reasoning, and irrelevant to the interest of literary truth. With this impression I conceived your readers would scarcely, on my part, have tolerated a reply *in detail* to the commentary of Mr. Cuming, as it involved no question of medical importance, and appeared almost exclusively a tissue of irritating remarks, evidently originating from that fruitful source, a wounded feeling. I experience no sentiment of regret in making the latter observation. I am no advocate for cynical

---

* Medical and Physical Journal, No. 74, Vol. xiii.

cal asperity, nor did I intend the tenor of my reflections to warrant the allegations of Mr. Cuming. In arrogating to myself the defence of Dr. Scott, I was animated by motives of a nature the most independant. I was aware of the urbanity of his private character, and of his ability and zeal in the cultivation of medical science. Regarding his observations as breathing the spirit of a disinterested advocate for truth, and also apprized of his aversion from *medical polemics*, I ventured to take up the gauntlet, bearing in mind the maxim of the great luminary of Roman eloquence: "*Hoc maxime officii est, ut quisque maxime opis indiget, ita ei potissimum opitulari.*"

I should however, from the motives previously assigned, and also from a general state of ill health, have declined any further agitation of the question, had I not conceived a few general suggestions necessary to evince, that my disposition bears little affinity with that of a *cynical Dictator*, and that I am in no shape ambitious, by displaying the petulance and asperity of a Scioppius, or a Dennis, to disprove the opinion of Antoninus, that we are in our nature more immediately prone to a conduct of a different description. — Ὁ ἄνθρωπος ἐυεργέτης πεφυκὼς.

I had trusted that my public communications, however feeble in their influence or defective in acumen of remark, might have been considered by Mr. Cuming as faint testimonials of my ambition to be useful in the walks of medical science; and I had presumed they would also have been regarded by inference as unequivocal evidence of my wish to cherish a spirit of liberal discussion in any controversy with my fellow labourers in that department; and never imagined he would deem me hostile to free and liberal enquiries into what regards the cultivation of an art that can only be improved by the united labors and zeal of such of its professors whose sole view is to render it honorable to themselves, and of utility to mankind in general.

I am, &c.

*Sunderland, June* 9, 1805. G. WILKINSON.

P.S. To find out the preparation of the cuprum ammoniacum, with which Mr. Cuming professes himself to be unacquainted, he must look into those Edinburgh Dispensatories preceding the date of his own; it is pretty well known as an excellent remedy in epilepsy, and is noticed by Dr. Odier of Geneva, in the Third Volume of the Edinburgh Medical Commentaries, and in Dr. Duncan's Medical Cases, published in Edinburgh, 1778.

